# Inhibition of Kirsten-Ras reduces fibrosis and protects against renal dysfunction in a mouse model of chronic folic acid nephropathy

**DOI:** 10.1038/s41598-019-50422-7

**Published:** 2019-09-30

**Authors:** Lucy J. Newbury, Jui-Hui Wang, Gene Hung, Bruce M. Hendry, Claire C. Sharpe

**Affiliations:** 1 Department of Inflammation Biology, School of Immunology and Microbial Sciences, King’s College London, London, UK; 2 Ionis Pharmaceuticals, Carlsbad, California 92010 USA; 3 Department of Nephrology, Cardiff University Medical School, Cardiff, UK

**Keywords:** Target validation, Chronic kidney disease, Renal fibrosis

## Abstract

Chronic Kidney Disease is a growing problem across the world and can lead to end-stage kidney disease and cardiovascular disease. Fibrosis is the underlying mechanism that leads to organ dysfunction, but as yet we have no therapeutics that can influence this process. Ras monomeric GTPases are master regulators that direct many of the cytokines known to drive fibrosis to downstream effector cascades. We have previously shown that K-Ras is a key isoform that drives fibrosis in the kidney. Here we demonstrate that K-Ras expression and activation are increased in rodent models of CKD. By knocking down expression of K-Ras using antisense oligonucleotides in a mouse model of chronic folic acid nephropathy we can reduce fibrosis by 50% and prevent the loss of renal function over 3 months. In addition, we have demonstrated *in vitro* and *in vivo* that reduction of K-Ras expression is associated with a reduction in Jag1 expression; we hypothesise this is the mechanism by which targeting K-Ras has therapeutic benefit. In conclusion, targeting K-Ras expression with antisense oligonucleotides in a mouse model of CKD prevents fibrosis and protects against renal dysfunction.

## Introduction

Chronic kidney disease (CKD) is one of only seven (of over 100) mortality-related diseases that are increasing in prevalence world-wide, and the only one for which there is no disease modifying treatment^1^. The need to develop drugs that have the potential to slow or prevent the progression of CKD towards end stage renal disease (ESRD) is therefore great. Fibrosis is the principal pathological process that underlies progression of CKD and results in an imbalance between extracellular matrix deposition and degradation leading to an accumulation of scar tissue and subsequent loss of normal renal parenchyma^2^. The only anti-fibrotic drugs currently available for the treatment of progressive fibrosis in any organ are nintedanib and pirfenidone, both licensed for use in idiopathic pulmonary fibrosis. Although the precise mechanisms of action of these drugs are unclear, they are both considered to be broad-spectrum receptor tyrosine kinase inhibitors^3^.

Ras monomeric GTPases are intracellular signalling molecules that direct upstream messages to downstream effectors involved in cell growth and differentiation^4^. Ras is central to the canonical signalling pathways of many receptor tyrosine kinase (RTK) ligands involved in fibrosis, such as EGF, PDGF and FGF, but in addition, is also activated by other pro-fibrotic ligands through non-canonical pathways, most notably TGF-β, EGF and Angiotensin-II^5–7^. Ras therefore acts as a convergent point in the signalling cascades downstream of the majority of cytokines that have previously been implicated as drivers of progressive fibrosis.

We have previously demonstrated that K-Ras is the key Ras isoform controlling human renal fibroblast proliferation^8^. In addition, when we knock down K-Ras expression using antisense oligonucleotides (ASO) in a rat model of unilateral ureteric obstruction (UUO), we reduce the degree of subsequent fibrosis by 60%^9^. Although UUO is a robust model of fibrosis, it does not provide a measure of renal function as an end point due to the remaining, fully functioning kidney.

In this study we have established a mouse model of chronic fibrosis associated with slowly progressive renal dysfunction, following 2 consecutive episodes of acute kidney injury (AKI) induced by folic acid administration. After recovery from the AKI, we show that K-Ras expression is increased in chronic folic acid nephropathy (CFAN) and that this can be reduced back to basal levels using mouse-specific K-Ras antisense oligonucleotides (ASO). This treatment results in a marked reduction in interstitial fibrosis, reduced Jag1 expression and normalisation of renal function.

## Results

### Consecutive episodes of acute kidney injury induced by low dose intravenous folic acid lead to accelerated chronic kidney disease in mice

**Figure 1:**
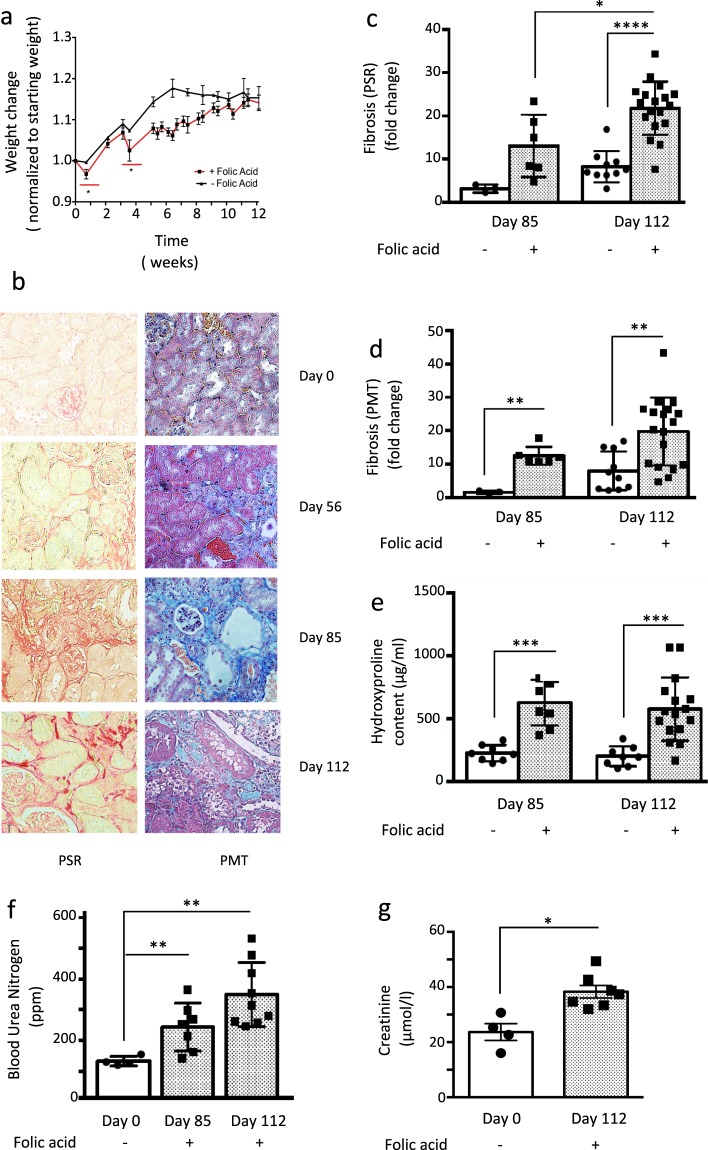
Repeated intravenous injections of folic acid are associated with accelerated fibrosis and CKD in mice. Male CD1 mice received 2 intravenous doses of 125 mg/kg of folic acid (FA) or normal saline (control). (**a**) Mice were weighed every 2–7 days throughout the model and their weights are shown. FA treated mice were weighed more often as a safety precaution. (**b)** Sections from mouse kidneys were stained with picrosirius red (PSR) at day 0 (no FA) and other time points post 1^st^ FA injection as indicated (representative images shown).In the PSR panels the red stain indicates the presence of collagen in the tissue. In the PMT panels the tissue matrix is indicated by the level of blue stain. (**c**) The PSR (collagen fibre) staining was quantified using NIS Elements software as described in methods. (**d**) Collagen staining was also quantified from picro-Mallory trichrome (PMT) stained sections using NIS Elements software as described in methods and (**e**) Collagen content was assessed by tissue hydroxyproline content. (**f**) Blood urea Nitrogen (BUN) and (**g**) Serum creatinine were also quantified. 8–16 mice per treatment group. Each point is the result for a single animal. Statistically significant differences are represented as follows here and in all figures: *p < 0.05, **p < 0.01, ***p < 0.005.

Folic acid is toxic to renal tubule cells and induces acute kidney injury (AKI) in rodents when administered intravenously at a high dose (250 mg/kg). In those animals that recover from the AKI, a slowly progressive interstitial fibrosis, associated with chronic kidney disease (CKD) ensues^10,11^. However, the mortality associated with the AKI phase in this model is high and the time taken for CKD to develop in those that survive is long (approximately 6 months). We therefore modified the model to reduce death in the acute phase and accelerate fibrosis in the chronic phase by injecting 2 lower doses of folic acid (125 mg/kg), separated by 21 days. Each injection was associated with weight loss in the affected mice during the period of AKI, which eventually recovered reaching comparable weights as the control group by week 10 (Fig. 1a). There were no deaths in the AKI period. Over the following 9 weeks the animals developed progressive fibrosis and CKD (Fig. 1b–g). Picrosirius Red (PSR) staining highlights the increasing deposition of collagen fibres in the interstitium of the kidney over time in representative kidney sections (Fig. 1b). This deposition is quantified using sections from all animals in Fig. 1c. Increased collagen deposition was also demonstrated and quantified using picro-Mallory trichrome staining (PMT) (Fig. 1d) and measurement of tissue hydroxyproline content (Fig. 1e). All three methods gave similar results. Renal function was also assessed demonstrating significant renal impairment by day 85 with a 2.5 fold increase in blood urea nitrogen (BUN) and a doubling of serum creatinine (Fig. 1f,g).

### K-Ras expression and activation are upregulated in chronic folic acid nephropathy and unilateral ureteric obstruction

**Figure 2:**
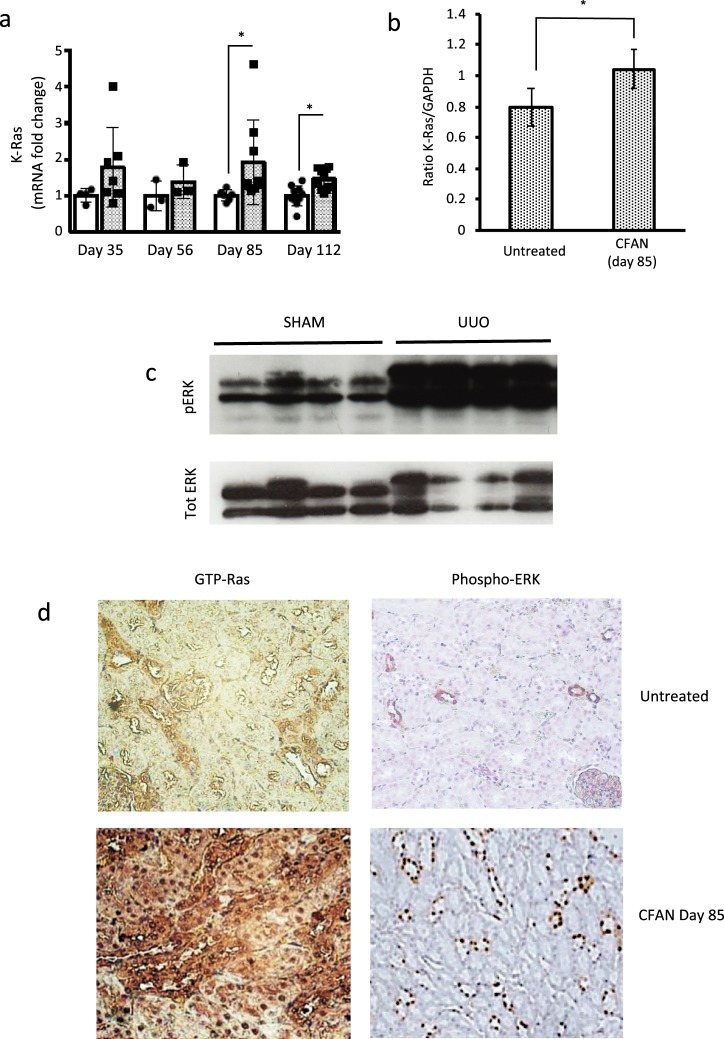
K-Ras expression is upregulated in chronic folic acid nephropathy (CFAN) and K-Ras activation is increased in CFAN and unilateral ureteric obstruction. Male CD1 mice received 2 intravenous doses of 125 mg/kg of folic acid (FA) or normal saline (control). Male Wistar rats underwent laparotomy followed by unilateral ureteric obstruction. Sham operated rats underwent laparotomy alone. (**a**) Total kidney mRNA was extracted from mice with chronic folic acid nephropathy (CFAN) (grey columns) or age-matched untreated controls (white columns) from different time periods and subjected to reverse transcription followed by quantitative PCR for K-Ras expression normalised to GAPDH. Each point or band represents a single animal, 8–16 animals in each treatment group. (**b**) Total Kidney lysate from CD1 mice with and without CFAN was subjected to immunoprecipitation (IP) followed by western blotting for K-Ras. The total cell lysate from the (IP) was then immunoblotted for GAPDH as an IP control. The densitometry ratio of K-Ras to GAPDH contains data from 4 untreated and 5 CFAN mice (Supplementary Fig. S2). (**c**) Total Kidney lysate from rats 16 days post UUO was subjected to western blotting for phosphorylated ERK 41/42 and compared with total ERK. A more complete image of this blot is shown in Supplementary Fig. S1. (**d**) Kidney sections from mice with CFAN (or untreated controls) were stained for GTP-bound (activated) Ras (left hand column) or phospho-ERK (right hand column). The tissue expression of these targets is seen as brown stain in the panels.

We have previously demonstrated that K-Ras expression is upregulated following unilateral ureteric obstruction^9^. Here we demonstrate that K-Ras expression is also upregulated in folic acid-induced nephropathy in the acute and chronic phases between 1.5 and 2 fold at the mRNA level and by circa 25% at the protein level (Fig. 2a,b and Supplementary Fig. S2). Although these increases in expression are modest in CFAN, they are associated with a marked increase in Ras activation, leading to increased GTP bound Ras and phosphorylation of ERK1/2, in both models (Fig. 2c,d and Supplementary Fig. S1). In contrast there was no upregulation of the other Ras isoforms N-Ras and H-Ras (data not shown).

### Reducing expression of K-Ras using antisense oligonucleotides reduces fibrosis in chronic folic acid nephropathy and protects against loss of renal function

**Figure 3:**
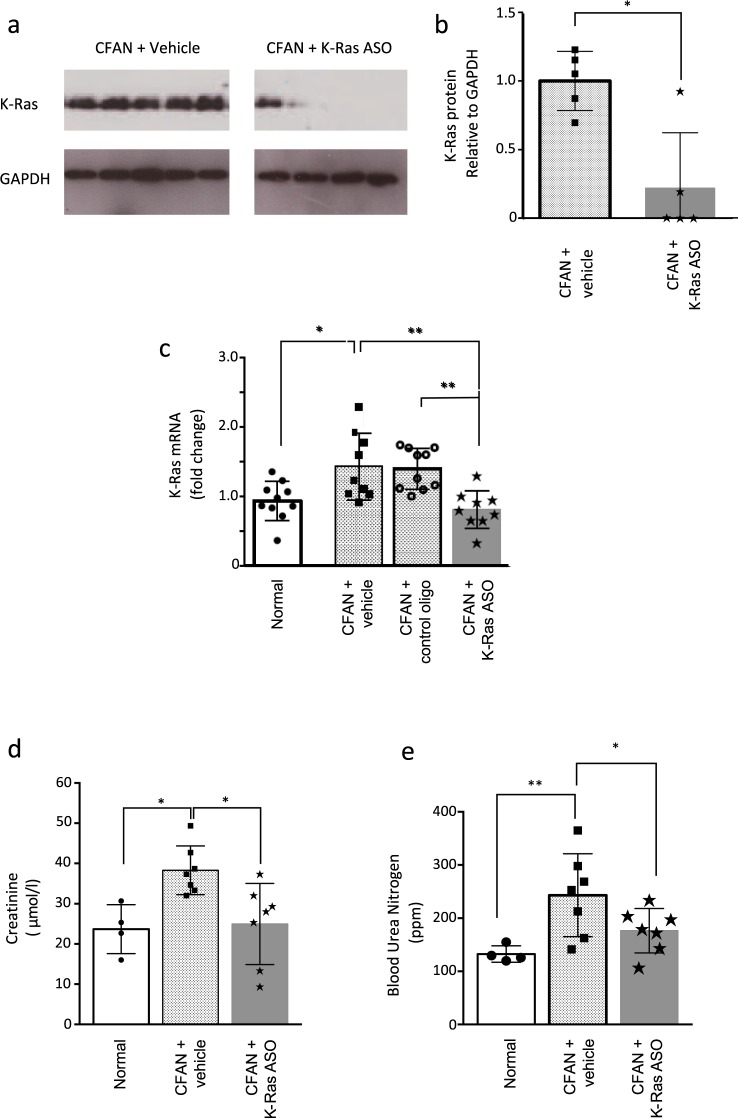
Mouse K-Ras ASO reduces K-Ras expression after folic acid administration and improves renal function. (**a**) Total Kidney lysate from CD1 mice with and without CFAN was subjected to immunoprecipitation (IP) followed by western blotting for K-Ras. The total cell lysate from the (IP) was then immunoblotted for GAPDH as a loading control. (**b**) Immuno-precipitated K-Ras expression was quantified using densitometry compared to GAPDH in the total cell lysate from the (IP) in separate blots as an IP control. (n = 5, *p < 0.05). (**c**) Total kidney mRNA was extracted from mice 112 days post 1^st^ FA injection and subjected to reverse transcription followed by quantitative PCR for K-Ras expression normalised to GAPDH. (**d**) Serum creatinine and (**e**) BUN were assessed in CFAN animals (85 days) treated or untreated with K-Ras ASO. Each point or band represents a single animal.

**Figure 4:**
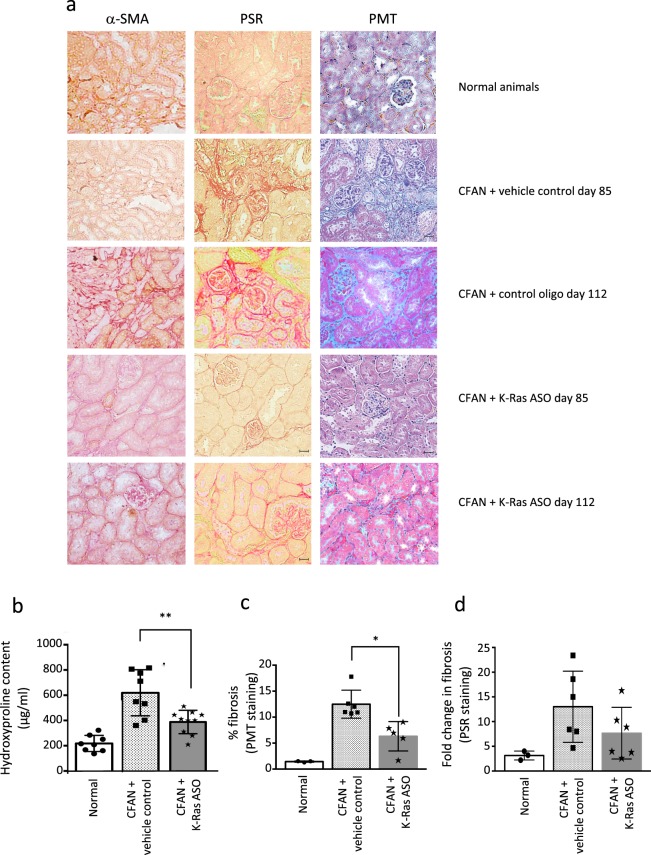
Mouse K-Ras ASO reduces fibrosis after folic acid administration. (**a**) Kidney sections from CFAN mice (with and without K-Ras ASO, vehicle or control oligo) or normal controls were stained for α-SMA, and fibrosis using PSR and PMT. Representative sections are shown. In the α-SMA panels the brown stain indicates the expression of the target. In the PSR panels the red stain indicates the presence of collagen in the tissue. In the PMT panels the tissue collagen content is indicated by the level of blue stain. (**b**) Tissue collagen (hydroxyproline) content was quantified in CFAN animals +/− K-Ras ASO (day 85) and fibrosis was quantified using: (**c**) PMT staining (day 112) and (**d**) PSR staining (day 85) using NIS Elements software as described in methods. Each point or band represents a single animal, n = 8 animals in each CFAN group.

One week following the second dose of folic acid, we treated mice with mouse K-Ras ASO to assess the impact of K-Ras downregulation on the development of fibrosis and renal dysfunction. Twice weekly subcutaneous injections of K-Ras ASO (50 mg/kg) were administered versus saline (vehicle) or a non-targeting control oligo. After 85–112 days mouse K-Ras ASO reduced K-Ras expression at both the message and the protein level by 50% and 75% respectively (Fig. 3a–c). This was associated with a 33% reduction in serum creatinine and a 29% reduction in BUN back down to levels in control animals, 8 weeks after disease induction (Fig. 3d,e). Although GFR was not formally measured, we believe the significant reduction in serum creatinine and BUN is consistent with better renal function in those animals treated with the K-Ras ASO. In keeping with this, treatment with mouse K-Ras ASO resulted in a 50% reduction in fibrosis as assessed by picosirius red (PSR) and trichrome (PMT) staining and a 30% reduction in kidney tissue collagen content compared to vehicle control (Fig. 4a–d). The active K-Ras ASO also reduced fibrosis as compared to the control oligo but this difference did not achieve significance (Supplementary Fig. S3).

### TGF-β-induced collagen expression in mouse proximal tubular cells is inhibited by K-Ras ASO administration

**Figure 5:**
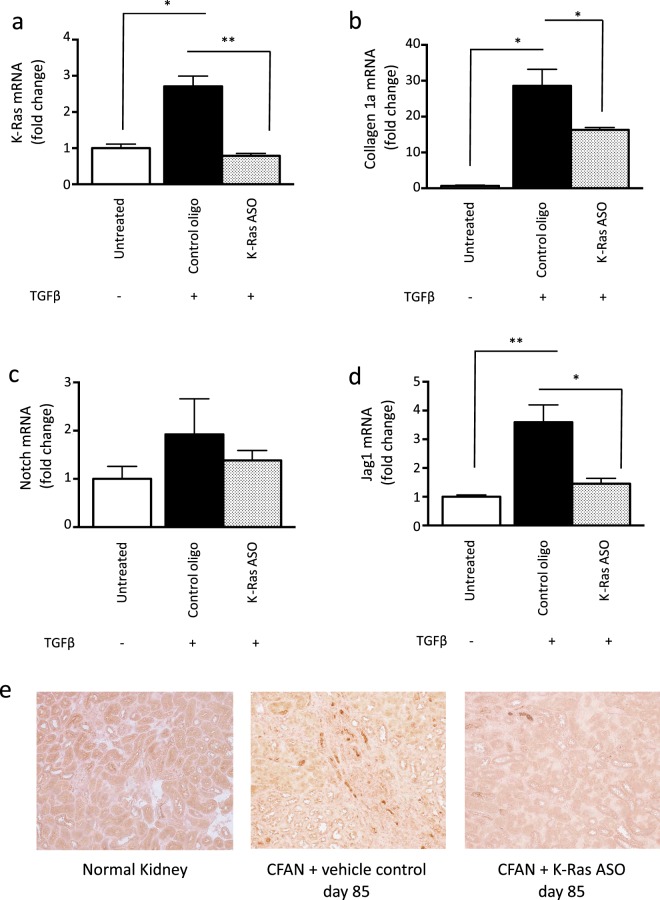
TGF-β increases K-Ras and collagen expression *in vitro*, which is prevented by mouse K-Ras ASO and TGF-β-induced Jag1 expression is inhibited by K-Ras ASO administration both *in vitro* and *in vivo*. Mouse proximal tubule cells (MPTECs) were cultured *in vitro* and treated with 10 ng/ml of TGF-β followed by mouse K-Ras ASO or a control oligo (each at 100 nmol/l). Total mRNA was then subjected to (**a**) K-Ras and (**b**) collagen 1a reverse transcription followed by QPCR. QPCR was also performed for (**c**) Notch1 and (**d**) Jag1. All QPCR data is normalised to GAPDH. (**e**) Immunostaining was performed for Jag1 on kidney sections from CFAN mice with and without K-Ras ASO treatment. The expression of Jag1 is shown as brown stain. Representative images are shown from a total of 4 animals per group.

To investigate the mechanism by which K-Ras inhibition prevents fibrosis, we treated mouse proximal tubular cells *in vitro* with TGF-β. This resulted in 2.8 fold increase in K-Ras expression and a 15 fold increase collagen expression. Pre-treatment with mouse K-Ras ASO completely prevented the increase in K-Ras expression and reduced the upregulation of collagen expression by 40% (Fig. 5a,b).

### TGF-β-induced Jag1 expression is inhibited by K-Ras ASO administration both *in vitro* and *in vivo*

Induction of renal fibrosis has previously been demonstrated to be dependent upon Jag1/Notch1 signalling. Indeed, overexpression of the intracellular domain of Notch1 in tubular epithelial cells is sufficient to induce aggressive renal fibrosis in mouse models^12^. Treatment of mouse proximal tubule cells with TGF-β confirmed upregulation of both Notch1 and Jag1. Concurrent treatment with mouse K-Ras ASO, had little impact on Notch1 expression but did prevent the upregulation of Jag1 mRNA, reducing the expression back down to that of untreated cells (Fig. 5c,d). This inhibition of Jag1 expression by mouse K-Ras ASO was also demonstrated *in vivo* using Jag1 immunostaining of kidney sections from CFAN animals (Fig. 5e).

## Discussion

In this study we describe a modification of the folic acid nephropathy model, which leads to aggressive interstitial fibrosis and renal impairment within 12 weeks following 2 consecutive episodes of acute kidney injury. We have demonstrated that K-Ras expression and activation are consistently upregulated throughout the chronic phase of this model and that this can be reduced back to normal levels by targeting K-Ras with mouse-specific antisense oligonucleotides. This treatment results in a 50% reduction in interstitial fibrosis and a near normalisation of renal function at the end of the model. Importantly the treatment is started on day 28, a week after the folic acid insults. The impact of K-Ras expression of the K-Ras ASO was significant compared to both vehicle control and control oligo and the phenotypic impact of this knockdown was persistent and consistently significant as compared to the vehicle control data. The K-Ras ASO phenotypic impact showed a trend to improvement when compared to the unrelated control oligo but this did not achieve significance due to the scatter of the *in vivo* data. Accordingly we are unable to unequivocally ascribe the mechanism of action of the therapeutic effects seen.

Previous *in vitro* and *in vivo* data support roles for the Ras GTPases in the genesis of renal fibrosis. We have demonstrated that renal fibroblasts express K-Ras and that this is required for normal proliferative responses^8^. The UUO and CFAN data presented here support the hypothesis that K-Ras ASO reduces fibrosis by reducing total K-Ras expression and hence reducing ERK phosphorylation^9^. Studies have also implicated both N-Ras and H-Ras in the fibrotic pathways of mouse renal cells^13,14^. N-Ras appears to be required for TGF-beta autoinduction and TGF-beta induced CTGF expression^5^. Data from mouse knockouts suggest that absence of H-Ras is protective in UUO-induced fibrosis^13^. In the current work we have demonstrated for the first time a therapeutic action of targeting K-Ras with ASO in terms of renal function improvement in an *in vivo* model of chronic fibrosis.

Ras monomeric GTPases are key signalling molecules, which play a pivotal role in embryological development and, when mutated, malignant transformation in many tumours. Indeed K-Ras knock-out mice exhibit an embryological lethal phenotype, whilst K-Ras mutants are responsible for 63% of all human pancreatic tumours and 33% of colorectal tumours^15^. It is therefore not surprising that a plethora of endogenous activators and inhibitors have evolved to tightly control the activity of these master regulators. One such endogenous inhibitor is RASAL1, a GTPase activating protein, which augments the natural ability of Ras to hydrolyse GTP to GDP causing inactivation Expression of this key Ras modulator has been previously shown to be downregulated in fibrotic renal tissue through hypermethylation of the CpG islands of the encoding gene and that inhibition of this methylation is protective against renal fibrosis^10^. This is also true for cardiac fibrosis where increased Ras activation drives fibrogenesis through activation of endothelial to mesenchymal transition^16,17^.

Although Ras isoforms are an attractive therapeutic target, no small molecules have been developed that effectively inhibit them directly^18^. Binding of the ASO to target mRNA results not only in steric inhibition of translation by the ribosomal complex but also in the induction of RNase H, which cleaves the 3′-O-P-bond of the RNA molecule. This mechanism of action theoretically provides 100% specificity for each Ras isoform, an unachievable goal for most conventional pharmacologic agents. In addition, this form of therapeutic has particular advantages when treating the kidney. When administered parenterally, ASO are filtered by the glomerulus and reabsorbed by proximal tubular cells achieving a concentration in the renal interstitium 80-fold over that seen in plasma^19^.

In conclusion we have demonstrated that K-Ras is upregulated in animal models of renal fibrosis and that preventing this upregulation can protect against progressive fibrosis and loss of renal function. Although targeting Ras has proven difficult over the years, antisense technology may offer a solution to targeting this elusive molecule in the treatment of chronic kidney disease. The data described here support both the generic concept of antisense as renal therapeutics and the specific targeting of K-Ras in the context of progressive renal fibrosis. Further work is warranted on the targeting of K-Ras and other Ras isoforms in renal fibrosis.

## Methods

### Oligonucleotides

Oligonucleotides were designed and produced by Ionis Pharmaceuticals, Carlsbad, California. They were 20-nucleotide, fully phosphorothioate “gapmers”. The five sugar residues on each end are 2′-O-methoxy-ethyl modified. The central 10 sugars are deoxyribonucleotides. All pyrimidine bases are 5-methyl substituted (T and me5-C). The K-Ras ASO was Ionis 487143.

### Cell culture

Mouse proximal tubular epithelial cells (MPTECs) were a gift from Dr Kenneth Hallows (University of Pittsburgh) and are derived from the S3 segment of the proximal tubule, originally derived from a Brinster Transgenic Mouse which contains the large T antigen of the SV40 virus [Tg(SV40E)Bri7]. Cells were grown in DMEM/F12 (1:1) medium (Life Technologies, Thermo Fisher Scientific, Waltham, MA USA) supplemented with: hydrocortisone (50 nmol), penicillin (100 U/ml), streptomycin (100 µg/ml) (PAA, GE Healthcare Ltd, Bucks, UK), amphotericin (260 µg/ml) and Insulin Transferin Selinate (ITS) (Gibco, Thermo Fisher Scientific) to 1X concentration. The cells were incubated in Techne incubators at 37 °C in 95% CO_2_ and 5% O_2_.

### Quantitative PCR

Total cellular RNA was extracted using a Qiagen RNeasy mini-kit (Qiagen, Crawley, UK). Tissue RNA was extracted using Trizol (Sigma Aldrich, St. Louis, Missouri, US) and homogenized with a potter homogenizer. The sample was then purified using a Qiagen RNeasy mini kit as above. Reverse transcription was undertaken using two kits, the omniscript kit (Qiagen, Cat:82840865) and RNA to cDNA high capacity kit (Applied Biosystems, Thermo Fisher Scientific). Quantative PCR was undertaken using Taqman technology (Life Technology; Thermo Fisher Scientific, K-ras: Mm00517494, H-ras: Mm00476174, Nras: Mm01308659, Cola1: Custom (576767B11), Jag1: Mm00496902, GAPDH: Mm99999915). The reaction was performed using the ABI Prism 7900HT machine (Applied Biosystems using TaqMan master mix as per manufactures instructions). Quantitative PCR mRNA data are presented normalised to the GAPDH signal as an internal control.

### Animal models

All experimental procedures were approved under provisions of the Animals (Scientific Procedures) Act 1986 and were performed under licence numbers PPL 70/6054 or PPL 70/7022 issued by the UK Home Office. The project was approved by the King’s College London Animal Welfare and Ethical Review Body. All methods and procedures were performed in accordance with the relevant guidelines and regulations.

#### Chronic folic acid nephropathy model (two-hit model)

Eight-week CD1 male mice were purchased from Harlan, UK and given one i.v. dose of folic acid (125 mg/kg) on day 1 followed by a second, similar dose on day 21. Each dose was followed by an episode of acute kidney injury associated with weight loss then recovery and the animals developed progressive renal fibrosis over the subsequent three months with no further intervention. There were no unplanned deaths in the acute phases and only one in the chronic phase. All mice were housed in batches of 4.

#### Unilateral ureteric obstruction

Unilateral ureteric obstruction (UUO) was undertaken in adult male Wistar rats obtained from Charles River, UK. Following a midline incision, the left ureter was isolated and ligated with two 6/0 silk ties. The abdomen was closed and buprenorphine (30μg/kg) was administered subcutaneously. Any first dose of treatment (oligonucleotide or vehicle) was also administered subcutaneously at this point. Animals undergoing the sham procedure underwent a full laparotomy and manipulation of the ureter without ligation. All animals were killed on day 16 after UUO.

### Immunoblotting

Proteins were extracted from cells or frozen tissue; the samples were run on SDS polyacrylamide gels of 8–15% depending on molecular weight of protein of interest and transferred on to nitrocellulose membrane. Following transfer the membranes were probed with primary antibodies for K-Ras (OP24, Calbiochem, EMD Millipore, MA, USA), GAPDH (Ma3374, Millipore), Jag 1 (sc-8303, Santa Cruz, CA, USA), pErK (4370, Cell Signaling, MA, USA), α-SMA (Ab5694, Abcam), Active Ras-GTP Monoclonal Antibody (26909, NewEast Bioscience) and subsequently probed with the appropriate secondary anti-mouse IgG or anti-rabbit IgG. Densitometry was performed using ImageJ software provided by NIH.

### Histology

Tissue was fixed in buffered formalin for 24 hours, embedded in paraffin, cut into 4 µm-thick sections and stained with H&E or special stains.

#### Special stains

Sections stained using the picro-Mallory trichrome (PMT) and picrosirius red (PSR) techniques were used to assess fibrosis. All sections were viewed under bright light. The degree of cortical fibrosis was scored using NIS-elements-Br 3.10, measuring the blue and red areas respectively in a randomized and blinded manner. The slides were quantified using a 200x quantification and the number of fields quantified depended on the stain in question. 10, 15, 20, and 25 fields per section were measured for the first three sections assessed from each stain. The average for each was compared, and when an increase in field number no longer changed the average by more than 10%, it was deemed an adequate number of fields for each section for that stain. The minimum number of fields per section sections for a stain was 10 with a maximum of 20.

#### Immunohistochemistry

Sections were mounted on polylysine-coated slides, baked at 40 °C overnight and deparaffinised. Antigen retrieval was performed by pressure-cooking in a citrate buffer (pH6) as required. Antibodies used were directed against modified oligonucleotides (supplied by Ionis Pharmaceuticals, rabbit polyclonal), α-smooth muscle actin (α-SMA) (A2547; Sigma-Aldrich Co. Ltd.). They were subsequently detected with a secondary ImmPRESS polymer reagents and DAB substrate (brown) (Vector Labs, California, US).

### Blood urea and nitrogen assay

Blood Urea Nitrogen (BUN) was measured using the MaxDIscovery Enzymatic Assay (Bio Scientific, Austin TX) in serum samples diluted by 1 in 5 with PBS. The assay was conducted as per the manufactures instructions.

### Serum creatinine assay

Creatinine was measured using a Creatinine enzymatic assay by Crystal Chem, Il, USA (Cat no. 80350). The assay was undertaken as per the manufactures instructions.

### Hydroxyproline assay

Hydroxyproline content was measured using the QuickZyme Bioscinces (Netherlands) total collagen assay (QZBhypro1). The samples were assayed as per the manufactures instructions.

### Statistical analysis

Statistics were performed using Graph Pad Prism 6 software. Both the ShapiroAWilk and D’AgostinoAPearson tests were used together to test for normal Gaussian distribution. Normally distributed samples were then analysed using a parametric test. When only two groups where compared this was followed with an unpaired T-Test. When 3 or more groups were compared a one-way Anova was conducted. This was followed by the post-hoc analysis of Tukey, allowing all groups to be compared with each other.

## Supplementary information

Supplementary Dataset 1

## Data Availability

The datasets generated and analysed during the current study are available from the corresponding author on reasonable request.
