# Supplementary material for: Inhibition of Kirsten-Ras reduces fibrosis and protects against renal dysfunction in a mouse model of chronic folic acid nephropathy
Source: Sci Rep. 2019 Sep 30;9:14010. doi: 10.1038/s41598-019-50422-7 (PMC6768870; doi:10.1038/s41598-019-50422-7)
Supplement: Supplementary file 1 — Supplementary Dataset 1 [file 41598_2019_50422_MOESM1_ESM.pdf]

Inhibition of Kirsten-Ras reduces fibrosis and protects against renal dysfunction in a mouse model of chronic folic acid nephropathy

Lucy J. Newbury[1], Jui-Hui Wang[1], Gene Hung[2], Bruce M. Hendry[1], Claire C. Sharpe[1]

Supplementary Figures

PhosphoERK & **Total ERK** in sham, UUO and ASO treated rat kidneys (day 16)

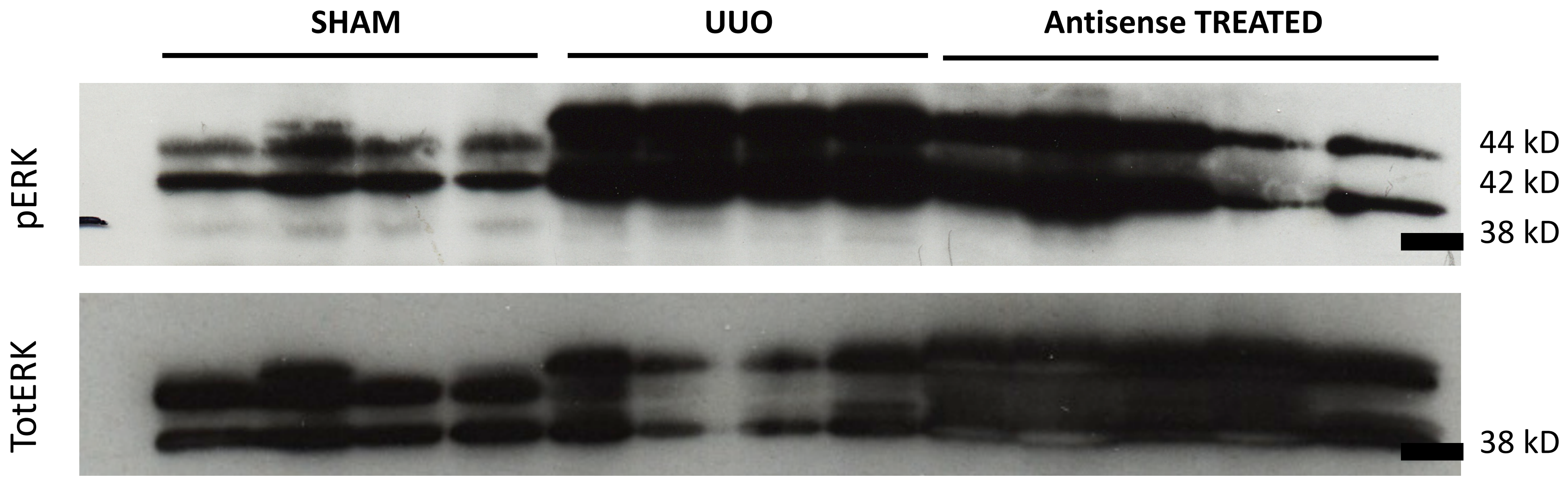

Figure S1: More complete view of the blot for ERK and pERK in the CFAN mice as described in Figure 2B

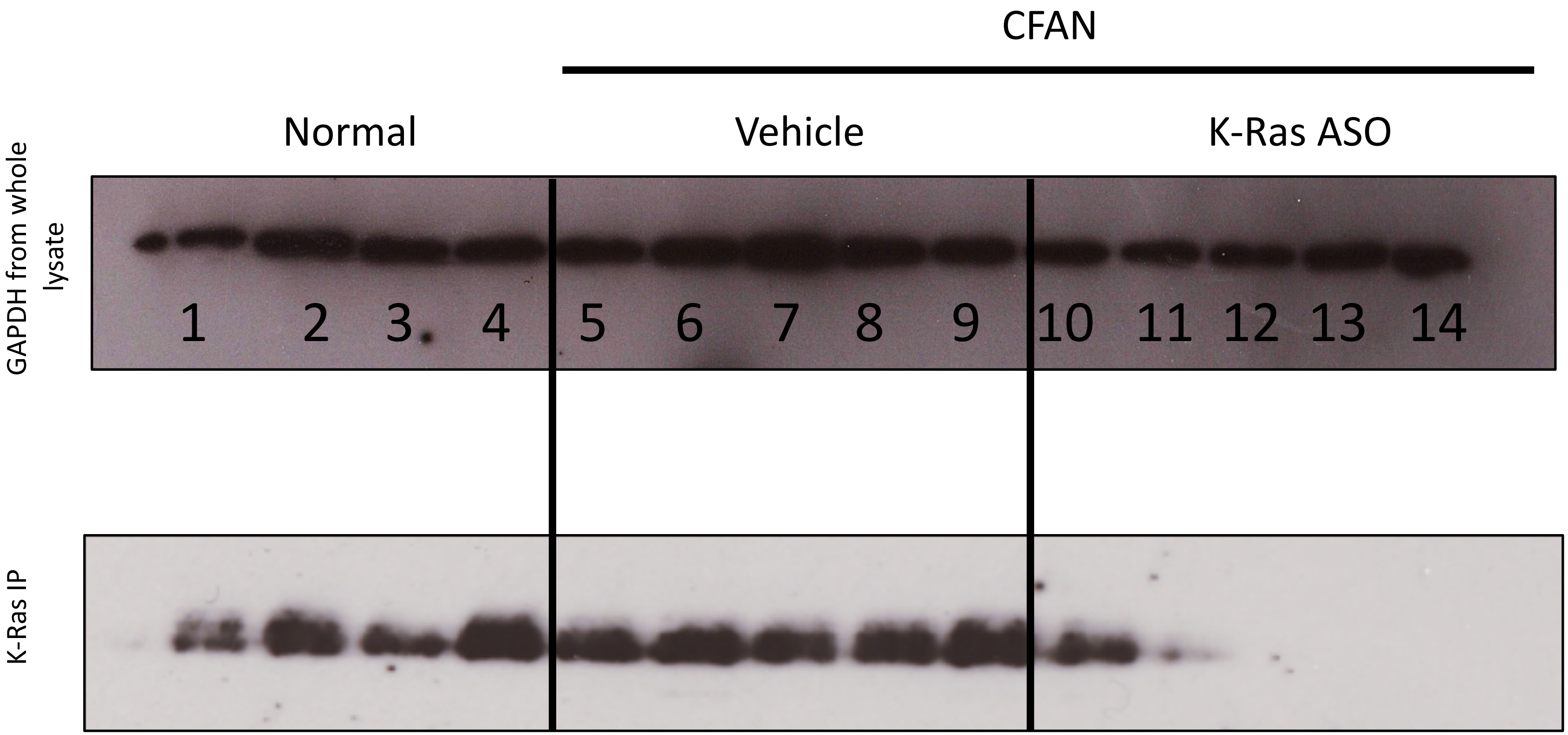

Figure S2:More complete view of the blot of K-Ras in the CFAN mice as described in Figure 3A.

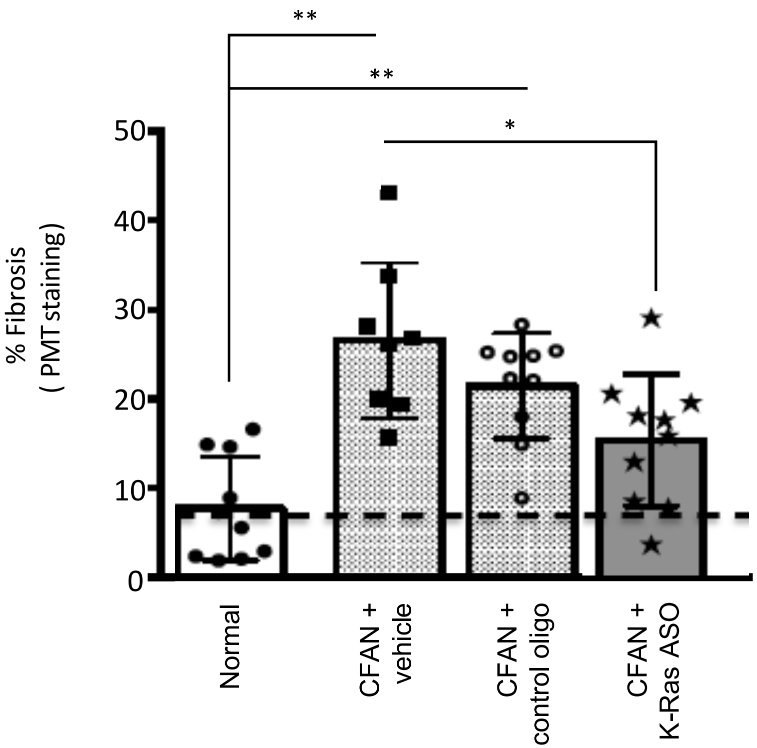

Figure S3: Data from the 112 day experiment testing K-Ras ASO against saline vehicle control and control oligo in the CFAN model. The graph shows the degree of fibrosis seen in the Mason's Trichrome stain quantified using NIS Elements software as described in methods. The active K-Ras ASO reduces fibrosis significantly compared to the saline vehicle controls in the CFAN disease animals. Each point represents data from a single mouse.
